# Simple-Structured OLEDs Incorporating Undoped Phosphorescent Emitters Within Non-Exciplex Forming Interfaces: Towards Ultraslow Efficiency Roll-Off and Low Driving Voltage for Indoor R/G/B Illumination

**DOI:** 10.3389/fchem.2020.630687

**Published:** 2021-03-15

**Authors:** Ting Xu, Ruichen Yi, Chunqin Zhu, Mingquan Lin

**Affiliations:** ^1^ Shenzhen Key Laboratory of Polymer Science and Technology, College of Materials Science and Engineering, Shenzhen University, Shenzhen. China; ^2^ Key Laboratory of Optoelectronic Devices and Systems of Ministry of Education and Guangdong Province, College of Physics and Optoelectronic Engineering, Shenzhen University, Shenzhen. China; ^3^ State Key Laboratory of Surface Physics and Department of Physics, Fudan University, Shanghai, China; ^4^ Jiangsu Key Laboratory for Carbon-Based Functional Materials and Devices, Soochow University, Suzhou, China; ^5^ Department of Electrical Engineering, City University of Hong Kong, Hong Kong, China

**Keywords:** indoor illumination, OLEDs, efficiency roll-off, simple structure, direct charge trapping

## Abstract

To meet the requirement of indoor R/G/B monochrome illumination a simplified OLEDs structure and fabrication process must occur. Herein, a design philosophy of low efficiency roll-off and simple-structure OLEDs incorporating R/G/B phosphorescent ultrathin non-doped emissive layers (EMLs) within non-exciplex forming interfaces a luminescent system by a direct charge trapping mechanism has been reported, which uses bis(2-methyldibenzo[f,h]-quinoxaline)(acetylacetonate)iridium(III) (MDQ)_2_Ir(acac), bis(3-phenylpyridin-e)iridium(III) (Ir(ppy)_3_), and bis(3,5-difluoro-2 -(2-pyridyl)phenyl-(2-carboxypyridyl) iridiumII) (Firpic) as R/G/B luminescent dyes, respectively. Although the recombination zone is narrow in the designed OLEDs, the efficiency roll-off of the designed OLEDs are unexpectedly slow, due to stable charge trapping of the emitters and are refrained from concentration quenching in relatively low current density, but the luminance meets the requirement of indoor lighting. With a low threshold voltage of 2.9/2.9/3.5 V, the designed R/G/B phosphorescent OLEDs show an efficiency roll-off as low as 7.6/3.2/4.3% for indoor luminance from 10 cd/m^2^ to 1,000 cd/m^2^, respectively. The perspective of R/G/B luminescent dyes on luminous efficiency, chromaticity coordinate drifts, efficiency roll-off, and direct charge trapping has been thoroughly studied. Therefore, our research may help to further develop ideal indoor lighting using a simplified undoped R/G/B OLEDs structure with simultaneous ultraslow efficiency roll-off, low threshold voltage, simplified fabrication process, low reagent consumption, and cost.

## Introduction

As an energy conserving and environmentally friendly healthy organic semiconductor illumination apparatus, organic light emitting diodes (OLEDs) have been widely applied in health lighting sources and displays due to their excellent properties since being invented by Tang and VanSlyke (1987) and Burroughes et al. (1990) based on small molecule and conjugated polymers, respectively. However, conventional OLED products are still rather expensive for consumers (Thejokalyani and Dhoble, 2014). Hence, it is imminently required to reduce the cost of products by simplifying the OLED structure and fabrication technology. OLEDs with a doping-free ultrathin emissive layer (UEML) have aroused research interest and have been developed rapidly due to their simple structure and easily fabrication which removes the need for doping and needs fewer sensors, which therefore reduces the requirement for equipment and lowers the cost of the OLEDs (Liu et al., 2016b; Xu L. et al., 2016). Two main aspects are promoting this field development. On the one hand, OLEDs with UEML have advanced rapidly with novel UEML materials (e.g., new complexes (Zhang et al., 2012), TADF (Zhang Q. et al., 2014; Hirata et al., 2015; Zhang et al., 2015; Ahn et al., 2019), and AIE (Liu et al., 2016a; Ruan et al., 2016). Simplified doping-free white OLED (Liu et al., 2014a) and yellow-orange OLED (Liu B. et al., 2015) are achieved based on a Pt(II)-based complex emitter that achieves rather low operating voltages and high power efficiencies (Wang et al., 2014). An efficient and concentration-insensitive metal‐free thermally activated delayed fluorescence (TADF) material was reported and applied in undoped OLEDs by the Adachi group (Zhang et al., 2015). Aggregation-induced emission (AIE), color tunable, efficient undoped OLEDs and good mechanochromic properties were confirmed (Ruan et al., 2016). On the other hand, a deeper understanding of device physics, photophysics (e.g., exciplex (Park et al., 2013; Seino et al., 2014; Shin et al., 2014), electroplax (Wang et al., 2004; Yang et al., 2009; Luo et al., 2017), excimer (Chen et al., 2016), or TADF (Lin et al., 2018) property), and novel device structure with efficient exciton harvesting (Schwartz et al., 2007; Wang et al., 2009; Cui et al., 2016) also play a key role in the development of OLEDs with UEML. The Cao group fabricated a novel white OLED with a doping-free process (Liu et al., 2014b; Liu et al., 2016b). The He group designed an inverted OLED with UEML (Liu et al., 2014), which was suitable for a display driven by an active matrix transistor backplane (Lee et al., 2008). The Ma group fabricated a highly efficient and simple monochrome OLED based on UEMLs within an exciton confinement energy band structure while a deeper understanding of the exciplex formation of TCTA and TmPyPB was not clearly pointed out (Zhao et al., 2013). The Ma group also designed a novel white OLED applied with an exciplex co-host and UEML in one device (Wang et al., 2016). The Lee group managed excitons distribution in TADF (Liu et al., 2015a) and white OLEDs (Liu et al., 2015b) by an exciplex energy transfer (Cui et al., 2015). We also developed a device design philosophy of OLEDs using mixed bulk (Xu et al., 2017b; Xu et al., 2019) or an interface exciplex forming host (Xu et al., 2017c), UEMLs (Xu et al., 2018b), double UEMLs (Xu et al., 2016b), or a tandem structure (Xu et al., 2018a) as a synergistic strategy (Xu et al., 2017a) toward a simplified OLED structure (Zhang et al., 2018) and fabricated the process without sacrificing device efficiency due to improving the energy transfer process. Nowadays, the indoor conditions of photovoltaic devices under low-illuminance conditions have been extensively researched as a specialized issue (Cui et al., 2017), (Xu et al., 2016a). Meanwhile, researchers rarely pay specialized attention to indoor lighting as the energy source of indoor photovoltaics. According to the international standard of the Commission Internationale de L'Eclairage (CIE): the lighting of indoor work places (ISO 8995:2002 CIE S 008/E:2001) (Rabich et al., 2017), and the corresponding brightness of interior illumination is generally from 10 to 1,000 cd/m^2^.

Herein, we designed and fabricated monochrome red, green, and blue phosphorescent OLEDs based on non-doped UEMLs to meet the requirement of indoor R/G/B monochrome illumination. Particularly, it is observed that the non-doped UEML-based OLEDs generally show very slow efficiency roll-off and low threshold voltage, which is fitting for indoor lighting in low current density with a brightness from 10 cd/m^2^ to 1,000 cd/m^2^. Moreover, the maximum power efficiency reached 5.766 lm/w at 177.8 cd/m^2^, 15.74 lm/w at 99.27 cd/m^2^, and 2.277 lm/w at 252.2 cd/m^2^ for the R/G/B monochrome OLEDs, respectively. The perspective of R/G/B luminescent dyes on luminous efficiency, chromaticity coordinate drifts, efficiency roll-off, and charge trapping mechanism has been thoroughly studied. Therefore, our work helps to develop ideal indoor lighting using a simplified undoped R/G/B OLED structure with simultaneous slow efficiency roll-off, low threshold voltage, simplified fabrication, low reagent consumption, and cost.

## Experiment Details

**FIGURE 1:**
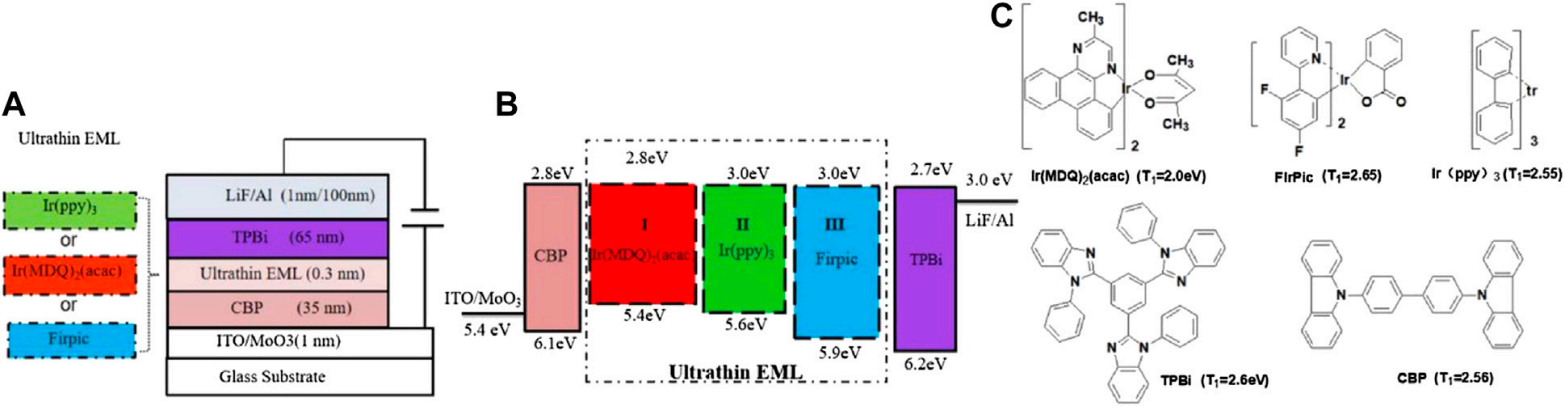
**(A)** Schematic device structure and **(B)** corresponding energy-level diagram of the devices considered in this work as well as **(C)** the molecular structure and triplet energies (T1) of the materials used.

All OLED devices were fabricated based on the same glass substrate pattern with a conducting indium-tin-oxide (ITO) anode and a sheet resistance lower than 20 Ω/square. The substrate fabrication was followed by a routine cleaning process (Xu et al., 2016a). The basic structure of the devices is a ITO/MoO_3_ (1 nm)/4′-bis(carbazol-9-yl) biphenyl (CBP) (35 nm) /Ultrathin emitter (0.3 nm)/1,3,5-Tris(N -phenylbenzimidazol -2-yl)benzene (TPBi) (65 nm)/LiF (1 nm)/Al (100 nm), in which the schematic parameter diagram and energy level diagram of the device structures are shown in Figures 1A, B, including MoO_3_ and CBP which act as the hole injection layer (HIL) and hole transporting layer (HTL). LiF and TPBi are used as the electron injection layer (EIL) and electron transporting layer (ETL). A 1 nm thick thermally evaporated MoO_3_ layer was deposited on top to achieve a high work function for the hole injection into CBP. The phosphorescent dyes in this study were bis(3-phenylpyridine)iridium(III) [Ir(ppy)_3_] for green in device A, bis(2-methyldibenzo[f,h]-quinoxaline) (acetylacetonate) iridium(III)[(MDQ)_2_Ir(acac)] for red in device B, and bis(3,5-difluoro-2 -(2-pyridyl)phenyl-(2-carboxypyridyl)iridium III (FIrpic) for blue in device C. Figure 1C shows the molecular structures of Ir(ppy)_3_, Ir(MDQ)_2_(acac), Firpic, TPBi, and CBP, respectively.

## Results and Discussion

Lots of researchers have proposed two primary mechanisms for the exciton process in OLEDs: host-guest energy transfer and direct charge trapping of the guest. Exciton formation occurs directly on the guest molecules, which play an important role in balancing charge injection. This direct charge trapping on the guest can be confirmed by the dependence of the drive voltage and electroluminescence spectrum on guest concentration (Holmes et al., 2003). The light emission mechanism of OLEDs with a mixed co-host emitting layer was also studied using an exciplex-type mixed co-host and an exciplex free mixed co-host (Song and Lee, 2015). The light emission process in the interface of exciplex-type OLED devices was dominated by energy transfer while the interface of non-exciplex type devices was dominated by a charge trapping emission mechanism (Wang et al., 2016). In this research, a design philosophy of low efficiency roll-off and simple-structure OLEDs incorporating R/G/B phosphorescent non-doped UEMLs within a non-exciplex forming interface luminescent system by a direct charge trapping mechanism was proposed. The device structures and the energy levels of the designed OLEDs are shown in Figure 1A,B.

**FIGURE 2:**
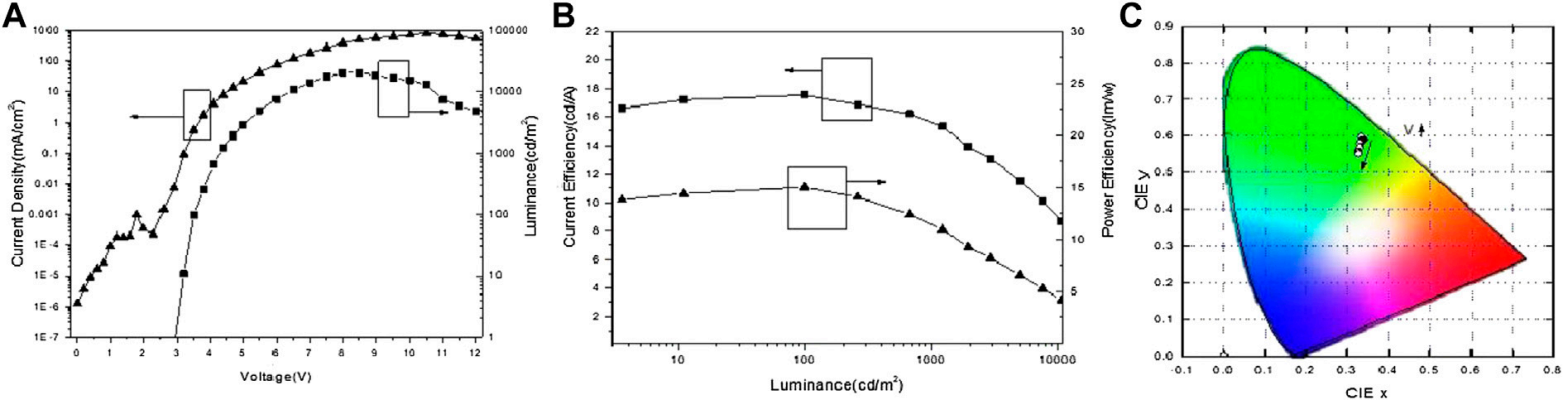
**(A)** Current density-voltage-luminance characteristics, **(B)** current efficiency-luminance-power efficiency characteristics, and **(C)** chromaticity coordinates of green OLEDs.

In working OLEDs, firstly, electrons and holes can be easily injected from the ITO anode and aluminum cathode under driving voltage, respectively. Then, electrons and holes go through ETL/HTL and meet to form excitons. There were some differences between devices A, B, and C. Under voltage from 2.9 to 4.9 V with a corresponding luminance from 10 cd/m^2^ to 1,000 cd/m^2^, the luminance of device A was linear to the logarithmic coordinate voltage of device A as shown in Figures 2A,B, and the current efficiency roll-off of device A was as low as 7.6%. As to the chromaticity coordinate of device A, the CIE_(x,y)_ was nearly unchanged from 2.9 to 4.9 V as shown in Figure 2C. This phenomenon is indicative of the direct charge trapping of Ir(ppy)_3_ as the luminescent dye of device A was quite effective in a low current density. Lastly, as the voltage increased after exceeding 5 V with a corresponding luminance of 1,000 cd/m^2^, the luminance of device A showed a gradual invariant with fast efficiency roll-off. There are three main reasons for this phenomenon. Firstly, the values of HOMO and LUMO of TPBi are closed to CBP as shown in Figure 1B, the energy level cannot block the leak current in a high current density, and carriers meet to form excitons in wide bandwidth HTL/ETL for inefficient lighting. Secondly, the direct charge trapping of Ir(ppy)_3_ as the luminescent dye of device A is saturated in high current density, while device performance is also saturated without assisting the energy transfer process (Forster energy transfer and Dexter energy transfer) in a non-exciplex system. Thirdly, the triplet state energy levels (T_1_) of CBP (2.55 eV) and TPBi (2.67 eV) could not be confirmed for luminescent dye Ir(ppy)_3_ (2.47 eV) in high voltage leading to drifting of excitons into a wide bandwidth HTL/ETL for inefficient lighting. Therefore, the chromaticity coordinate CIE_(x,y)_ of device A was remarkably changed following the direction as the voltage increased after exceeding 5 V as shown in Figure 2C.

**FIGURE 3:**
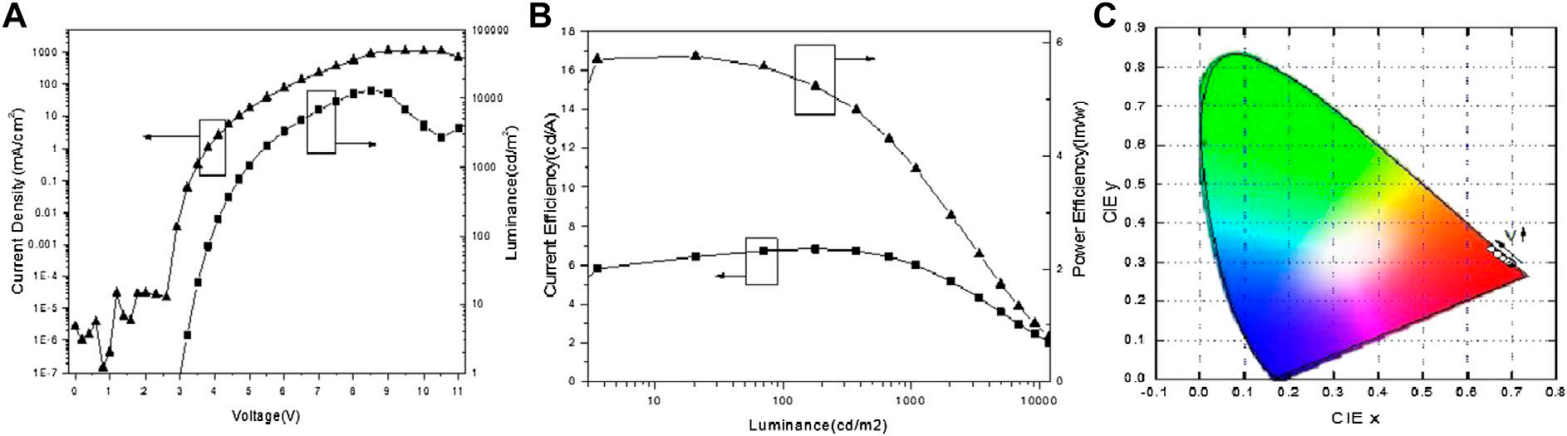
**(A)** Current density-voltage-luminance characteristics. **(B)** Current efficiency-luminance-power efficiency characteristic. **(C)** Chromaticity coordinate of red OLEDs.

**FIGURE 4:**
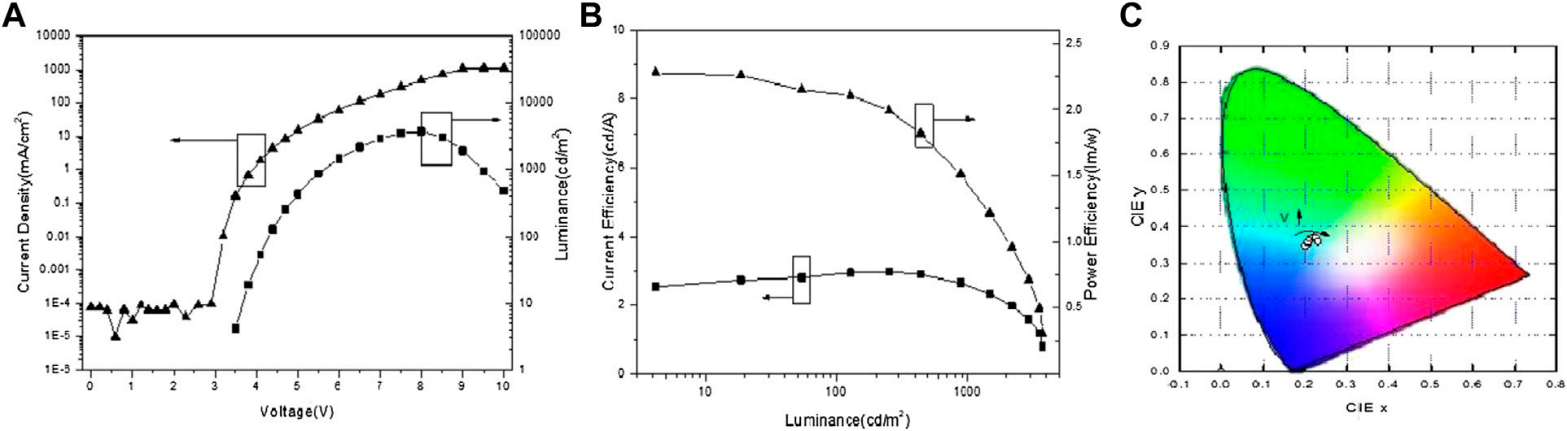
**(A)** Current density-voltage-luminance characteristics. **(B)** Current efficiency-luminance-power efficiency characteristic. **(C)** Chromaticity coordinate of blue OLEDs.

As seen in Figure 3A and Figure 4A, under low voltage with corresponding luminance from 10 to 1,000 cd/m^2^, the luminance of devices B and C were linear to the logarithmic coordinate voltage of devices B and C. The current efficiency roll-off of devices B and C were as low as 3.2 and 4.3% in as shown in Figure 3B and Figure 4B, respectively. The CIE_(x,y)_ of devices B and C were nearly unchanged under low voltage while the CIE_(x,y)_ of devices B and C changed remarkably following the direction as the voltage increased after exceeding 5–6 V as shown in Figure 3C and Figure 4c.

Thanks to the equal thickness of devices A, B, and C, the J-V-L curves of the phosphorescent dyes were different as shown in Figures 2A,B, Figures 3A,B, and Figures 4A,B. In low working voltage, there was no significant difference for J-V among devices A, B, and C implying that exciton quenching or the nonradiative decay of excitons was greatly suppressed by the direct charge trapping of this simple UEML within the non-exciplex interface TPBi/CPB. However, the efficiencies of the OLEDs with UEML were affected by the phosphorescent dyes with different T_1_ levels. The case of CPB or TPBi as the host with exciton formation as the guest should be similar to this OLED with non-doped UEML by direct charge trapping. The utilization of the non-doped UEML structure yields high efficiencies for the green OLED with Ir(ppy)_3_ as the UEML, while the efficiencies of the red and blue OLEDs with (MDQ)_2_ Ir(acac) and Firpic were not as high as the green dye on account of relatively low PLQY, which was similar in the host-guest doping system. The non-doped UEML was constructed by introducing a 0.3 nm thin layer of pure phosphorescent dyes between the HTL and ETL layer. The maximum current efficiency reached 15.74 lm/w at 99.27 cd/m^2^, 5.77 lm/w at 177.8 cd/m^2^, and 2.28 lm/w at 252.2 cd/m^2^ for the green, red, and blue monochrome OLEDs, respectively.

To further explore the working mechanism of the designed OLEDs with UEML in high current density, Supplementary Figure S1 shows the electroluminescence (EL) spectra of the non-doped OLEDs with UEML for the R/G/B phosphorescent dyes at the higher voltage of 8 V. It can be seen that device A with Ir(ppy)_3_ and device B with Ir(MDQ)_2_ (acac) show pure dye emission due to the high T_1_ level of CPB and TPBi confirming direct charge trapping excitation in the relatively lower T_1_ level of Ir(ppy)_3_ or Ir(MDQ)_2_ (acac) in high current density. In device C with FIrpic, it is interesting that the EL spectra peak around 380 nm showed the characteristics of host materials intramolecular luminescence in TPBi (Zhang T. et al., 2014) rather than in CBP (Park et al., 2011), which indicates exciton diffusion into TPBi. This may have been due to ETL occurring intensely or due to the charge carrier recombination zone in TPBi, and then radiative decay which emitted light into TPBi in a primary way. There was also a secondary peak around 450 showing the dye emission of FIrpic due to direct charge trapping of FIrpic to form exciton, but the EL of FIrpic was much weaker than the emitted light in TPBi due to the relatively low efficient exciton energy transfer from TPBi (Jang et al., 2011) to FIrpic as shown in Supplementary Figure S1. Moreover, there was a wide peak around 700 nm which shows the characteristics of inter-molecular overlay of the red-shifted excimer of phosphorescence (Kalinowski et al., 2007). Low direct charge trapping of FIrpic, exciton diffusion to ETL, and low efficient exciton energy transfer from TPBi to FIrpic led to the low efficiency of device C in high current density.

The J-V-L characteristics, the CE-L-PE characteristics, and the chromaticity coordinate of the device A are shown in Figures 2A–C, separately. Similarly, Figure 3 and Figure 4 show the corresponding performance of device B and device C. The turn-on voltages (V_on_) (which are defined as a luminance of 1 cd /cm^2^) of devices A and B were as low as 2.9 V, and device C was 3.5 V as summarized in Table 1. The maximum current and power efficiency (η_c max_, P_max_) of (17.5 cd/A, 15.7 lm/W), (6.9 cd/A, 5.8 lm/W), and (2.98 cd/A, 2.3 lm/W) were obtained in devices A, B, and C, respectively. And the maximum brightness of devices A, B, and C were as high as 20,700, 13,210, and 3,740 cd/m^2^, respectively. The phosphorescence dyes have different capabilities to trap the charge carriers directly leading to different efficiencies in the OLEDs with UEML.

**TABLE 1:** A summary of OLEDs with non-doped UEML.

Device	V_turn-on_ (V)	L_max_ (cd/m^2^)	η_c max_ (cd/A)	η_c_@500 nit (cd/A)	P_max_ (lm/W)	Roll-off (%) (10∼1,000 cd/m^2^)
A	2.9	20,700	17.5 (5.1%)	16.2	15.7	7.6%
B	2.9	13,210	6.9 (2.3%)	6.7	5.8	3.2%
C	3.5	3,740	2.98 (1.3%)	2.9	2.3	4.3%

**FIGURE 5:**
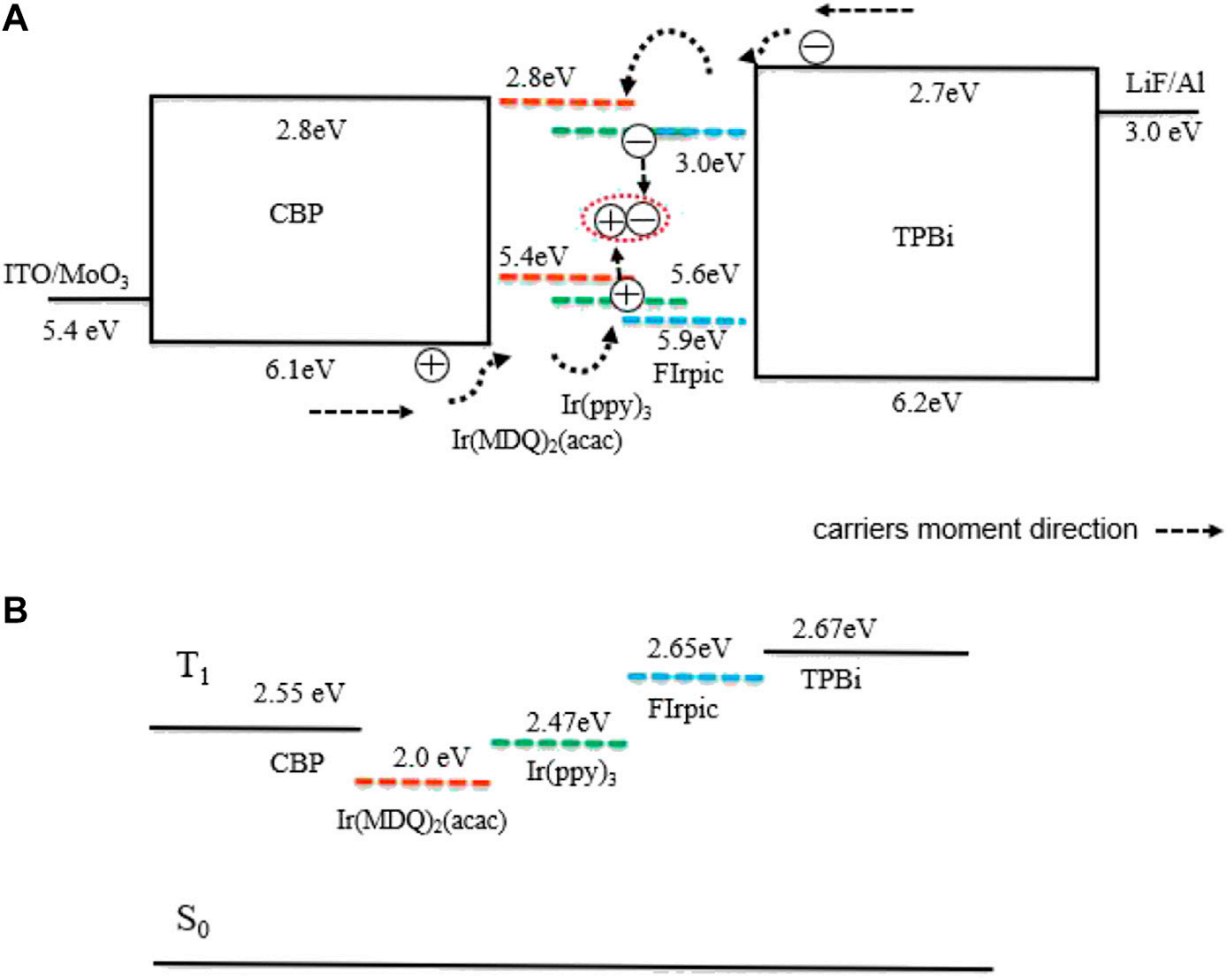
**(A)** Energy diagrams and carrier transfer route of proposed OLEDs. **(B)** Triplet energy level T1 of CBP, TPBi, Ir(MDQ)2(acac), Ir(ppy)_3_, and FIrpic.

In low working voltage, the power efficiency of the blue dye OLEDs with non-doped UEML was as high as the red one. The utilization of this kind of non-doped UEML within non-exciplex forming interfaces can be appropriate for R/G/B dyes. All the monochrome R/G/B OLEDs based UEML show relatively high efficiency and ultraslow efficiency roll-off in low driving voltage, the non-doped UEML structure within a non-exciplex forming interface has broad adaptability for most phosphorescent dyes with ultraslow efficiency roll-off in low driving voltage. As there is relatively few reports on non-exciplex forming interfaces as direct charge tapping structures that have such broad adaptability for phosphorescent dyes with ultraslow efficiency roll-off in low driving voltage, our technical route offers a brief scheme to achieve indoor lighting requirements. Further, in order to clearly describe the work mechanism of this device structure, a physical model of exciton dynamics in OLED with non-doped UEML is proposed in Figure 5.

The higher turn on voltage for device C suggests that the excitons from direct charge trapping are not efficient comparing with devices A and B. As depicted in Figure 5A, some of the carriers would enter into the UEMLs as deep as 3 nm (Zhao et al., 2012), and then would recombine in the organic layers, while the direct charge carrier recombination on phosphorescent dyes would generate dye emission due to the thin nature of non-doped UEML. These excitons may either transport their energy from ETL or HTL to the EML. The efficiency of OLEDs with Ir(MDQ)_2_ (acac) was lower than that of OLEDs with Ir(ppy)_3_ since the LUMO energy level of Ir(MDQ)_2_ (acac) was nearly equal to the LUMO energy level of CBP and TPBi. There was also no doubt that the capability of Ir(MDQ)_2_ (acac) to direct charge trapping was weaker than that of Ir(ppy)_3_ in the same thickness. From the OLED-based UEML, the energy transport process from the ETL/HTL to the EML was much more than that of exciton diffusion in device A and device B. Meanwhile, as depicted in Figure 5B, thanks to the higher T_1_ exciton between the transporting layers of T_1_ of CBP and TBPi, the excitons generated were effectively confined in the UEML for the Ir(MDQ)_2_ (acac) and Ir(ppy)_3_ dyes. As to the blue OLED with FIrpic, the efficiency of the OLEDs with FIrpic was lower than that of OLEDs with Ir(ppy)_3_ since the T_1_ energy level of FIrpic was higher than that of CBP. Although the recombination zone is narrow in the designed OLEDs, the efficiency roll-off of the designed OLEDs are unexpectedly slow mainly due to the stable charge trapping of the emitter and avoiding concentration quenching in relatively low current density for the requirements of indoor lighting. With a low threshold voltage of 2.9/2.9/3.5 V, the designed R/G/B phosphorescent OLEDs show an efficiency roll-off as low as 7.6/3.2/4.3% in the indoor luminance intensity range from 10 to 1,000 cd/m^2^, respectively.

## Conclusion

In summary, we succeeded in applying a series of R/G/B ultrathin non-doped phosphorescent dye layers into non-exciplex forming interfaces as a cost-effective method to realize OLEDs with ultraslow efficiency roll-off and low driving voltage to satisfy indoor R/G/B illumination requirements in low driving voltage. This device structure shows great superiority in easy fabrication, low-cost, and high-efficiency when compared to compact fluorescent lamps due to the highly simplified ultrathin and undoped emitters. This technical route has high value for the future of indoor solid-state lighting applications.

## Data Availability

The original contributions presented in the study are included in the article/Supplementary Material, further inquiries can be directed to the corresponding author.
