# Supplementary figures and images for: Simple-Structured OLEDs Incorporating Undoped Phosphorescent Emitters Within Non-Exciplex Forming Interfaces: Towards Ultraslow Efficiency Roll-Off and Low Driving Voltage for Indoor R/G/B Illumination
Source: Front Chem. 2021 Mar 15;8:630687. doi: 10.3389/fchem.2020.630687 (PMC8005586; doi:10.3389/fchem.2020.630687)

**Supporting Information:**


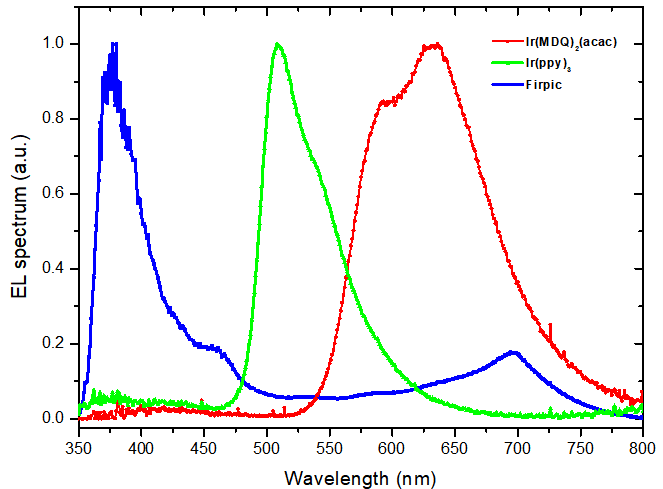


SI **Fig. 1** EL spectrum of R/G/B OLED device at 8 V

Supplement: Supplementary file 1 [file table1.docx]
